# How effective are films in inducing positive and negative emotional states? A meta-analysis

**DOI:** 10.1371/journal.pone.0225040

**Published:** 2019-11-21

**Authors:** Luz Fernández-Aguilar, Beatriz Navarro-Bravo, Jorge Ricarte, Laura Ros, Jose Miguel Latorre

**Affiliations:** 1 Psychology Department, University of Castilla-La Mancha, Albacete (Spain); 2 Applied Cognitive Psychology Unit, Medical School, University of Castilla La-Mancha, Albacete (Spain); 3 Unidad de Investigación, Fundación del Hospital Nacional de Parapléjicos, Albacete (Spain); Victoria University of Wellington, NEW ZEALAND

## Abstract

Meta-analyses and reviews on emotion research have shown the use of film clips to be one of the most effective methods of mood induction. Nonetheless, the effectiveness of this method when positive, negative and neutral emotional targets are studied under similar experimental conditions is currently unknown. This comprehensive meta-analysis included only studies that implemented neutral, positive and negative mood inductions to evaluate the effectiveness of the film clip method as a mood induction procedure. In addition, several factors related to the films, sample and experimental procedure used, the number of emotional categories, for example, or the number of film clips watched, were included to study their influence on the effectiveness of this mood induction procedure. Forty-five studies were included with 6675 participants and 12 possible moderator variables according to the sample and the research procedure. Our findings suggest that film clips are especially powerful in inducing negative mood states (Hedges’ g for valence = -1.49 and for arousal = -1.77) although they are also effective inducers of positive mood states (Hedges’ g for valence of = . -1.22 and for arousal = -1.34). Additionally, this meta-analysis reveals that variables, such as the number of emotional categories or the type of stimulus used to measure the baseline, should be considered.

## Introduction

Over the last three decades, interest in the study of emotions has increased notably, focusing both on the construct itself and its interaction with other concepts such as cognition, behavior, personality and physiology [1–3].

Controlled mood induction enables us to better know, understand and manage our emotions. For this reason, much effort has been made in emotion research to create systems that artificially elicit emotional changes. Numerous *Mood Induction Procedures* (MIPs) have been developed to generate positive, negative and neutral mood states (see [4, 5] for a revision, [6]). Some procedures use autobiographical information, such as autobiographical memories [7, 8], while other procedures use written texts, such as Velten MIP [9] and the reading of fragments of books [10]. A number of procedures use acoustic stimuli, such as imagination MIPs e.g., [11, 12], the International Affective Digitized Sound System (IADS [13]) and music MIPs (e.g., [14]). Pictures are used in others procedures, such as the International Affective Pictures System (IAPS [15]). Procedures have also been implemented involving the manipulation of the expression, thought or behavior of the participants, for example, the Facial Action Coding System FACS [16] and social interaction of success or failure [17, 18]. Finally, audiovisual materials, such as virtual reality [19, 20] and films [21], have been utilized in certain procedures.

Although all these systems seem capable of eliciting positive, negative and neutral mood states, they also present several limitations [5, 6, 22]. First, one of their main limitations is that of demand characteristics, which refer to participants’ being aware of the purpose of the experiment and shaping their responses accordingly. Second, another limitation of some MIPs is the lack of standardization, as is the case of autobiographical recalls, imagination MIP or behavioral inductions [22, 23]. Third, potential priming or cognitive priming can occur, instead of eliciting emotions, in the Velten MIP, for example, or the reading of texts [24]. Fourth, when the goal is to elicit negative emotions, it is of great importance to control the ethical limitations. For example, in the case of real-life manipulations and autobiographical recalls, traumas might be evoked [25–27]. Finally, another limitation may be the obtaining of discrete emotions or avoiding the attenuation of the mood induction, when, for example, the length of exposure increases, as may happen in the case of the IAPS [15].

The substantial use of audiovisual materials to induce emotions has evidenced that it is one of the most easy-to-use techniques in the laboratory [28]. One of the main reasons for its success is that film clips can generate a dynamic context using stimuli that are similar to those in real life, but without the ethical problems that may arise when manipulating emotions [29]. Film clips are also effective in eliciting discrete emotions and have a greater effectiveness in prolonged maintenance of both subjective and physiological changes in emotion [25, 30]. Furthermore, this procedure has been greatly standardized, with sets of film stimuli being used in different settings and with different populations e.g., [2, 31]. Nevertheless, the film method also has drawbacks. The film clips that are used in emotional induction studies are frequently from popular films and thus the camera angles, lighting, settings and/or characters may vary from one clip to another. In addition, viewing films requires high cognitive demand and, therefore, may not be suitable for working with certain populations (e.g., individuals with cognitive impairment) [21]. Finally, there may be demand characteristics in the use of this technique, although this greatly depends on the specificity of the instructions (e.g., [32]).

The systematic reviews and meta-analysis of mood induction published to date clearly demonstrate the effectiveness of film clips in inducing emotions. [4–6, 22]. However, these reviews have not addressed important questions about films. In 1996, Westermann and colleagues published the first systematic quantitative review on the effectiveness of MIPS, in which they analyzed the effectiveness of 11 MIPs in inducing positive and negative mood. The authors analyzed the effects of MIPS when different kinds of manipulation check measures are used, and also assessed the effects of MIPs depending on gender, occupation and demand characteristics. The results of their study revealed that film clips exhibited the largest effect size on the induction of positive and negative emotions. However, it is worth noting that the authors classified studies published between 1975 and 1990, while the most significant increase in interest in the use of audiovisual sets actually occurred in the following decade. Indeed, the main sets of film stimuli currently used in research were developed after 1990 (e.g., [26, 28, 33, 34, 35]). Subsequently, the meta-analysis conducted by Lench and colleagues in 2011 examined the effectiveness of 10 MIPs inducing discrete emotions.These authors studied whether happiness, sadness, anger, and anxiety elicit changes across cognitive, judgment, experiential, behavioral, and physiological systems. However, they did not control for the potential moderators of each of the MIPs. For example, they coded whether participants completed the mood induction alone or in a group, but only provided a general finding for the set of MIPs studied. Their meta-analysis suggested that mood induction tends to be more effective when the participants are alone but we cannot know whether the finding would be similar in the case of film MIPs. Both studies [5, 22] focused on the general features of MIPs without going into the details of each procedure.

Previous reviews have found that most MIPs are more effective in inducing negative mood states than positive ones, although this difference is not considered to be significant in the case of film MIPs [5, 6]. However, it is not currently known whether this effect would be maintained if specific features of film clips (e.g., number of film clips used, baseline measure, conditions, etc.) were controlled for. To the best of our knowledge, no reviews have controlled for the influence of the specific features of films in mood induction. For example, no reviews have examined whether the studies assess the emotional targets (positives, negatives and neutrals) using the same experimental design or whether this effect is maintained when the same experimental design is used to induce both negative and positive emotions.

A meta-analysis is a meticulous method of reviewing scientific evidence but the use of the technique without applying critical evaluation may result in a biased work [36, 37]. Thus, it is crucial to control for the lack of uniformity in the different study designs, as methodological heterogeneity impacts on the conclusions drawn from the review. For this reason, in the present study, we selected only studies with a similar experimental design and which included positive, negative and neutral film clips in the MIP. The characteristics of the stimuli and the measuring instruments used in the studies also had to be similar for all three types of mood induction (positive, negative and neutral).

Previous research has shown that self-reported quantitative measurement of mood state provides stronger effects than other response systems, such as cognitive, behavioral or physiological responses [22]. Consequently, for the present review, we selected studies that used self-reported experience to measure the induction capacity of film clips. Self-reported experience is the subjective interpretation of mood states and is measured by means of questionnaires based on an emotion model. Most questionnaires measuring emotional response are based on the dimensional affect model [38], but there are others that use the discrete model of emotions [39]. Our work includes self-reports based on both models of emotion as they both provide important information that helps understand the structure of the emotion system. Discrete emotion models classify emotions by their functions and their universal character in primary emotions, including a range of positive and negative emotions with different developmental functions [40]. Thus, each emotion has a concrete representation (e.g., disgust, surprise, happiness or sadness) [41, 42]. For example, disgust and fear are classified as negative emotions and are considered to have different functions, disgust being associated with rejection and fear with protection. The dimensional model proposes the existence of two dimensions to define emotions: valence, or the pleasant-unpleasant dimension; and arousal, or level of intensity of emotional reaction [38, 43]. Valence and arousal are interrelated but independent dimensions [44], and are widely used as a measure of emotional states in research [45, 46]. Furthermore, some authors support the idea that measures of emotional response reflect dimensions rather than discrete states e.g., [47]. In order to obtain an adequate sample size, we chose to include studies based on dimensional and discrete perspectives. When the studies selected included more than one emotional model to assess the emotional response, we only selected those using dimensional self-reports.

The main objective of this review is to provide practical information on potential methodological moderators. We assess the potential influence of film-related variables associated with the mood induction. What variables facilitate effective induction of positive and negative mood states? To date, several questions remain unsolved. For example, is the technique more effective in group or individual sessions? Participants may be more likely to use distraction as a form of emotional regulation when in a group [22] but, on the other hand, emotional contagion through facial expressions is common in groups [48]. Is the effectiveness of films for inducing emotions ensured in both general and clinical populations? The presence of emotional disorders is associated with difficulty in responding adequately to emotionally significant stimuli [49, 50]. For example, bipolar disorder is linked to an increase in self-reported positive emotion using film MIP [51] and dysphoria is associated with the inability to maintain positive emotions [52]. Other important variables to consider are gender, age and sample origin (university community vs. others). Many studies have suggested that women report stronger negative emotions and rate neutral stimuli more positively than men [53, 54], while other studies suggest that men report stronger anger than women [55]. Therefore, the proportion of male and female participants could influence MIP, while age might also impact on the strength of the MIP. Previous studies suggest that older adults tend to report lower negative emotions than young participants [56]. Regarding the origin of the sample, university participants tend to respond more to demand characteristics than other community members [22]. There are also unanswered questions about the experimental procedure and film set variables. Previous studies have not explained whether it is more effective to use a single film clip or to use several film clips. Studies that use more than one clip usually study more than one emotional category. Some studies have only used one emotional target (e.g., sadness) and others studies more than one. Using a large number of stimuli to elicit different emotions may result in respondent fatigue [57] or the physiological transference of one emotion to another [58]. For this reason, it might be thought that the strength of induction depends on the number of films, emotional categories or conditions used in the laboratory. How clips are presented (random or fixed order) could also influence the effectiveness of the induction. Finally, there are other potential methodological moderators. For example, no previous reviews have studied the type of neutral stimulus used. The most commonly used neutral stimuli to establish the baseline are (a) watching a film clip with neutral content; (b) watching a clip from a nature documentary; and (c) watching a shapes screensaver. Other, less common stimuli used include participants closing their eyes for a short time or taking several deep breaths. The form and content of these control stimuli may generate unwanted differences in the results [59]. Lastly, no previous reviews have studied how the audio of the film clips impacts on the strength of induction. For example, verbal film clips and music film clips may add intensity to the emotional experience (see [34]). The emotion model of the self-report instruments was also included.

As previously mentioned, the main objective was to provide practical information on potential methodological moderators. We aimed to determine the most suitable experimental conditions to improve the effectiveness of film clips in inducing positive and negative emotions in the laboratory. To this end, we selected studies that evaluated the capacity of film clips to induce emotions by means of neutral, positive and negative emotional targets. Based on the samples and the procedures used in these studies, the following issues were addressed in the current meta-analysis: (a) differences in induction using positive and negative stimuli; (b) influence of factors or moderating variables on the study design (affective reactions by sample and affective reactions by research procedure).

## Method

### Literature search

All the studies were selected by means of a search through PsycINFO, Medline (PubMed), Psicothema, Scopus and Web of Science from inception to October 2017. The criteria used in the search of journal articles were the combination of the terms “emotion” OR “mood”; -AND “induction” OR “elicitation” OR “manipulation”; -AND “film” OR “movie”. Furthermore, the studies identified were back-referenced. Published reports were also considered and articles written in English and Spanish were both included. To determine which studies were useful to our work, we reviewed titles and abstracts, the screening of which was carried out independently by LF & JR (Kappa intercoder reliability = .93). In case of disagreement, the full text was read and discussed until a consensus was reached.

### Inclusion/exclusion criteria

Studies were accepted for the meta-analysis if they met the following criteria: (a) the study investigated both positive and negative emotions and neutral state; (b) the participants’ affective state was measured with a self-report instrument; (c) self-reports were based on the dimensional or the discrete model of emotion; (d) the results were reported with sufficient detail to allow calculation of effect sizes.

Studies were excluded from the meta-analysis if: (a) they used films with an aim other than that under study (e.g., using film clips to measure empathy levels); (b) if the stimuli were not used to induce any of the emotional targets in the present research (e.g., surprise); and (c) if they used combined MIPs (e.g., film clips and the Velten method).

After database extraction, hand-searching for studies potentially overlooked or absent from the databases was performed by screening the references of all retrieved articles. The review was executed following meta-analysis (PRISMA) guidelines [60].

### Categorization of variables

In accordance with our research interests, several rules were established for the categorization of variables. All characteristics included in this review were coded according to information available in the published texts. In line with the first research question in this meta-analysis, in which we attempt to describe differences between the induction of positive and negative emotions, we have classified the emotions, taking into account both the dimensional model of emotion and the discrete emotion model. Specifically, the different emotional states were grouped into two single categories according to the emotional tone. Positive emotional tone includes positive valence (dimensional emotion model) and the emotions of joy, amusement, happiness, contentment, tenderness and elation (discrete emotion model). Negative emotional tone includes negative valence (dimensional model of emotion) and the emotions of disgust, sadness, anger and fear (discrete emotion model). Moreover, the arousal level was also categorized for neutral, negative and positive mood inductions.

Surprise was excluded from the analysis because its emotional valence is unclear. In the literature, surprise has been treated as both a pleasant (e.g., [61]) and an unpleasant emotion (e.g., [62]).

For the present meta-analysis, we selected works that studied the emotional response to positive, negative and neutral stimuli using a similar experimental design. The response to neutral stimuli was used as the baseline measure. Including a baseline measure allows the strength of the mood induction to be calculated for both negative and positive mood states.

Regarding the second research question, we examined several variables related to the characteristics of the studies to test for potential moderator variables. The potential moderators were determined according to the sample population and the research procedure. As regards the characteristics of the sample, we coded the average age of participants and included whether participants were young or older adults. In view of possible gender differences, the proportion of females was coded. Whether participants were college students vs. members of the community (e.g. participants recruited through advertisements in local newspapers) was coded because the university community is the most common sample in psychology research. We also coded whether the sample consisted totally or partially of clinical individuals, taking into account clinical or neurological pathology, such as depression or dysphoria

Regarding the research procedure, several potential moderators were coded. First, the emotional model was considered. Some studies have analyzed the strength of film clips by emotional dimensions and others by discrete emotions. We coded whether the studies used one or the other emotional model. Second, when the studies assessed the emotional dimensions, we coded whether these measured only one dimension (valence or arousal) or both. Although all the studies included in the present review measured the valence dimension, only 46% of the studies included a measure of the arousal dimension. Third, we recorded the variables according to the research procedure and film set variables. We coded whether participants completed the MIP in group or in individual sessions. If this was not specified, we assumed that participants completed the emotion induction alone. The number of conditions in the experimental design were included in the review. We coded whether all participants watched all film clips (one condition) or whether they were divided into three groups and each of them watched neutral, positive or negative film clips (three conditions). In addition to the above, we recorded whether participants watched the film clips according to high or low arousal in positive and negative mood induction (five conditions). With regard to film clips used as stimuli to induce moods, the number of clips viewed in each study varies greatly. The study with fewest film clips used just one and the study with the largest number included 60 clips. For that reason, we coded the number of film clips watched by each participant. The number of emotional categories elicited also varies. Thus, we coded whether participants were induced to a single emotion category (positive, negative or neutral) or more than one. Some studies included only one category to induce a negative emotion (e.g., sadness) and one category to induce a positive emotion (e.g., amusement), while other studies included more than one category for both mood inductions (e.g., sadness and anger for negative induction; amusement and happiness for positive induction). The influence of the order in which film clips were shown was also included. We coded when the participants watched the clips in a similar (fixed) order and when they watched the clips in a different order (random order). Some studies included only visual clips and others included audiovisual clips. We coded, then, whether the films were shown with or without sound. Finally, we coded the type of neutral stimulus used to establish participants’ basal state. According to the studies selected for this review, we classified the neutral stimulus in four categories: popular film clips without emotional content, dynamic color shapes (screensaver), a combination of both previous neutral stimuli and other less common stimuli (e.g., rest period).

### Calculating effect sizes

With the data reported in each study, we used the Comprehensive Meta-Analysis program (Version 2; CMA; [63]) to estimate effect sizes for affective reactions generated by film induction. First, we attempted to explain heterogeneity by including moderator/independent variables. We assessed for the possible presence of heterogeneity across studies by using the *Q* test for heterogeneity and the *I*^*2*^ index, which describes the percentage of heterogeneity. Heterogeneity was considered low, moderate or high, based upon values of 25%, 50% or 75%, respectively. Second, when these statistics reported heterogeneity in effect sizes, we conducted analyses to calculate Hedges’ *g* under the random-effects model, which takes within-study variance, sampling error and between-studies variance into account. We used Hedges’ effect size as the main effect size measure, considering 0.2 a small effect size, 0.5 medium, and 0.8 large. The effect sizes were expected to be negative. For the studies using the dimensional model to measure the valence of emotions evoked by negative stimuli (n = 27), we inverted the scores so that the effect sizes would have the same sign in all the studies. To this end, we inverted both responses to the neutral stimulus and the responses to the negative stimulus using the following formula: lower limit of the scale–value of the stimulus + upper limit of the scale. This is because we calculated effects sizes from the neutral mean score of each study to identify the effectiveness of the induction method. We compared this neutral mean score with the negative mean score and the positive mean score. To do this, we obtained the mean negative affect scores in the neutral condition and compared them with the mean negative affect scores in the negative condition. In the same way, we obtained the mean positive affect scores in the neutral condition and compared them with the average positive affect scores.

When a study used different emotional scales, we selected the scale corresponding to the dimensional model of emotion to analyze the valence and arousal. Effect sizes were calculated from means and standard deviations and when these data were not available in the journal articles, we contacted the authors.

Publication bias was measured using Egger’s test. We used a funnel plot to generate a graphic representation of this potential publication bias. The main issue in publication bias is that not all completed studies are published. Studies with larger effects sizes are more likely to be accepted for publication. Taking into account that the meta-analysis can overestimate the true effect size because it may be based on a biased collection of studies, it is important to assess the likely extent of the bias. We used Egger's Test to assess the publication bias.

Various meta-regressions were performed to establish which variables could have an effect on heterogeneity. We also calculated the *Q*_*R*_ (to find whether the effect size varied across subgroups), the *I*^*2*^ (percentage of variation in the effects observed which reflects variance in true effects rather than sampling error), and the *R*^*2*^ (percentage of variance in the real effects explained by the model). The possible moderator variables were the following: age; percentage of female participants in each study; type of sample (whether the sample consisted totally or partially of individuals with emotional disorders, or older adults, or individuals from a university environment); the emotional model used (dimensional or discrete model of emotion); the emotional dimension measured in case of the dimensional model (valence, arousal or both); type of session (group or individual); sound (whether the film clips were shown with or without sound); presentation of clips (random or fixed order); type of neutral stimulus used (nature documentary, shapes screensaver, a clip from a popular film, a combination of film clips and screensaver, or other less common stimuli); number of conditions (whether participants were exposed to stimuli from a single category or whether they watched fragments from various or all of the categories); number of films viewed by each participant; and number of discrete categories considered in each emotional dimension used in the experiment (i.e., a film reflecting tenderness and another reflecting enjoyment would be considered two positive categories).

## Results

### Description of the studies

**Fig 1:**
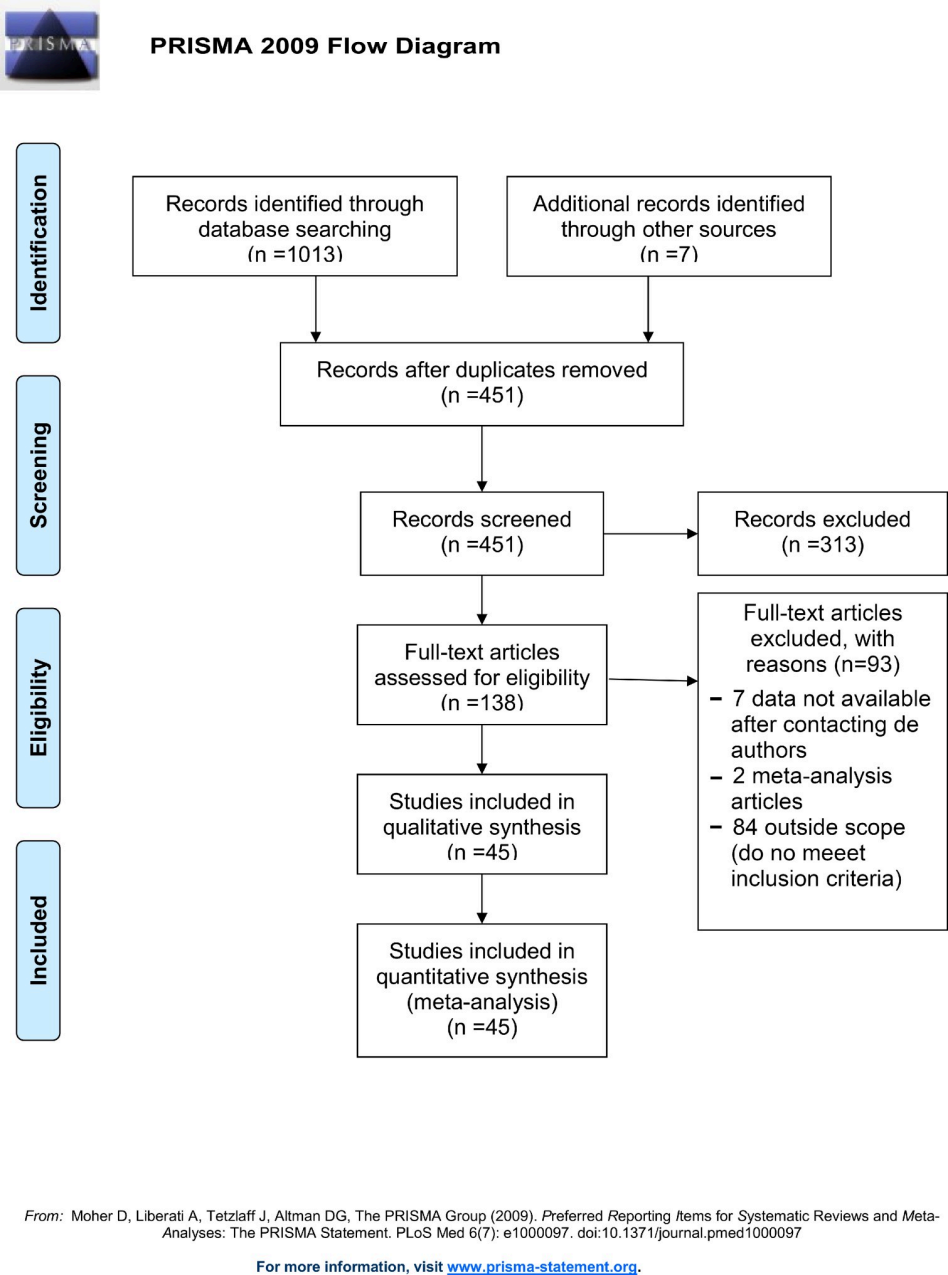
Flow chart.

After the initial screening procedures, we obtained 451 citations from the databases. Of these, 313 were discarded because they did not meet the inclusion criteria after reviewing the abstracts. The remaining 138 citations were assessed and reviewed for eligibility in more detail. We excluded 93 full-text articles (7 due to unavailability of data after contacting the authors, 2 meta-analysis articles, and 84 not meeting the inclusion criteria). Finally, 45 studies were included in the present meta-analysis (Fig 1).

After an exhaustive search from inception to the present, we found that the earliest study meeting the criteria to be included in our meta-analysis was published in 1993, meaning that the studies selected in this review were all published between 1993 and 2017. The 45 studies covered 6,362 non-clinical participants and 313 participants with emotional disorders. Most of the studies used undergraduate samples (n = 29) and most participants were females (66.87%). The weighted mean age in the samples was 33.88 years, with some studies including children (n = 1) and older adults (n = 4). All studies used films as the method to evoke positive, negative and neutral states. Table 1 details the characteristics of the populations and the procedures used in each selected study.

**Table 1:** Reviewed studies included in the meta-analysis.

	Sample					Procedure								
Studies	*(N)*		Mean age	Proportion women (%)	Population	Emotional dimension	Emotional model	Film features	Neutral stimulus	Session	Screening method	Audio	Measure	Contrast
	Non-clinical	Clinical												
Beaudreau et al. (2009) [64]	AD (30) OL (30)		21 73	67,75 66.70	UC Other	VA, AR	DEM	C(1) F(10) NNC(3) NPC(1)	SHA	IN	RAN	S	PFQ	Between subjects
Boyano & Mora (2015) [65]	AD (569)		21.89	65.89	UC	VA	DIEM	C(3) F(1) NNC(1) NPC(1)	DOC	IN	RAN	S	PANAS	Within subject
Carvalho et al. (2012) [25]	AD (113)		21.56	66.37	UC	VA, AR	DEM	C(1) F(13) NNC(2) NPC(1)	DOC	GR	RAN	S	SAM	Within subject
Carvalho et al. (2016) [66]	AD (125)		25.62	58.40	UC	VA, AR	DIEM	C(3) F(3) NNC(1) NPC(1)	DOC	IN	RAN	S	PANAS ES	Within subject
Cerully & Klein (2010) [67]	AD (187)		18.89	67.40	UC	VA	DIEM	C(3) F(1) NNC(1) NPC(1)	DOC	IN	RAN	S	PFQ	Within subject
Chou et al. (2007) [68]	AD (98) OL (90)		20.30 67.72	56.12 56.66	Other	VA	DEM	C(3) F(1) NNC(1) NPC(1)	FIL	IN	RAN	S	DIMS	Within subject
Connell et al. (2017) [69]	AD (140)		28.05	73.57	Other	VA	DIEM	C(1) F(3) NNC(1) NPC(1)	SHA	IN	FIX	S	DIES	Within subject
Curby et al. (2012) [70]	AD (90)		20.90	73.11	UC	VA, AR	DEM	C(3) F(1) NNC(1) NPC(1)	Other	IN	RAN	S	AFFECT GRID	Within subject
Dawkins et al. (2007) [71]	AD (29)		24.36	68.80	Other	VA	DIEM	C(1) F(12) NNC(1) NPC(1)	FIL	GR	RAN	S	PFQ	Between subjects
Fajula et al. (2013) [61]	AD (19) OL (19)		26 76	68.42 68.42	Other	VA	DIEM	C(1) F(6) NNC(4) NPC(1)	DOC	IN	FIX	S	DES	Between subjects
Falkenstern et al. (2009) [31]	AD (86)		19	66.12	UC	VA	DIEM	C(3) F(2) NNC(1) NPC(1)	FIL/SHA	GR	RAN	S	ERF	Within subject
Fernández et al. (2011) [29]	AD (127)		29.30	72.40	UC	VA, AR	DEM	C(1) F(8) NNC(4) NPC(2)	FIL/SHA	IN	RAN	S	SAM	Within subject
Fernández et al. (2012) [72]	AD (123)		29.20	73.98	UC	VA, AR	DEM	C(1) F(10) NNC(4) NPC(2)	FIL	IN	RAN	S	SAM	Within subject
Fredrickson & Branigan (2005) [73]	AD (104)		UNK	66	UC	VA	DIEM	C(3) F(1) NNC(2) NPC(2)	SHA	IN	RAN	S	ERF	Within subject
Gabert-Quillen et al. (2014) 62]	AD (304)		18.90	55.92	UC	VA, AR	DEM	C(1) F(9) NNC(4) NPC(3)	DOC	GR	RAN	S	PFQ	Within subject
Gilman et al. (2017) [74]	AD (784)		19.98	76	UC	VA	DIEM	C(1) F(6) NNC(2) NPC(2)	DOC	IN	RAN	S	PFQ	Within subject
Gómez et al. (2005) [75]	AD (75)		24	48.68	UC	VA, AR	DEM	C(5) F(2) NNC(2) NPC(2)	Other	IN	RAN	S	SAM	Within subject
Gruber et al. (2011, Experiment 1) [51]	AD (24)	BD (23)	35.46 39.13	52 77.80	Other	VA, AR	DIEM	C(1) F(6) NNC(1) NPC(1)	FIL	IN	RAN	S	PANAS AFFECT GRID	Between subjects
Gruber et al. (2014) [49]	AD (23)	BD (23)	35.24 39.13	52 73.9	Other	VA	DIEM	C(1) F(3) NNC(1) NPC(1)	FIL	IN	RAN	S	PANAS AFFECT GRID	Between subjects
Hagemann et al. (1999) [33]	AD (42)		24.60	52.28	UC	VA, AR	DEM	C(1) F(13) NNC(3) NPC(1)	DOC	GR	RAN	WS	DIMS	Within subject
Hewig et al. (2005) [59]	AD (38)		22.30	55.26	UC	VA	DIEM	C(1) F(20) NNC(4) NPC(1)	FIL	GR	RAN	WS	DIMS	Within subject
Hinojosa et al. (2017) [76]	AD (22)		23	72.72	UC	VA, AR	DEM	C(1) F(6) NNC(1) NPC(1)	FIL	IN	RAN	S	SAM	Within subject
Jenkins & Andrewes (2012) [34]	AD (54) OL (55)		30.07 59.53	48.14 50.90	Other	VA	DIEM	C(1) F(60) NNC(4) NPC(2)	FIL	IN	RAN	WS	VAS	Between subjects
Jurásová & Špajdel (2013) [77]	AD (173)		21.60	71.67	UC	VA, AR	DEM	C(1) F(11) NNC(1) NPC(1)	FIL/SHA	IN	RAN	S	SAM	Within subject
Koval et al. (2013) [50]		DS (95)	19.06	62.62	UC	VA	DIEM	C(1) F(10) NNC(2) NPC(2)	FIL	IN	FIX	S	PA/NA	
Koval et al. (2015) [78]	AD (200)		18.32	55	UC	VA	DIEM	C(1) F(10) NNC(2) NPC(2)	FIL	IN	FIX	S	PA/NA	Within subject
Koval et al. (2016) [79]	AD (100)		20.77	86	UC	VA	DIEM	C(1) F(10) NNC(2) NPC(2)	FIL	IN	FIX	S	PA/NA	Within subject
Lenton et al. (2013, Experiment 1) [80]	AD (112)		20.23	79.46	UC	VA	DIEM	C(3) F(1) NNC(1) NPC(1)	DOC	IN	RAN	S	PANAS	Within subject
Maffei et al. (2014) [81]	AD (32)		23.34	50	UC	VA, AR	DEM	C(1) F(20) NNC(4) NPC(1)	FIL	IN	RAN	S	PLEIN	Between subjects
McMakin et al. (2009) [52]	AD (26)	DY (21)	19.77 20.13	54.80 81.30	UC	VA	DEM	C(1) F(3) NNC(1) NPC(1)	SHA	IN	RAN	S	CONAR	Between subjects
Overbeek et al. (2012) [82]	AD (83)		32.78	49.39	Other	VA, AR	DEM	C(1) F(7) NNC(4) NPC(1)	FIL	IN	RAN	S	EMOEX	Within subject
Palfai et al. (1993) [83]	AD (72)		UNK	UNK	UC	VA	DIEM	C(3) F(5) NNC(2) NPC(2)	FIL	GR	RAN	S	SIMC	Within subject
Rottenberg et al. (2002) [84]	AD (33)	DD (72)	32.30 33.40	69.30 66.70	Other	VA	DIEM	C(1) F(4) NNC(2) NPC(1)	DOC	IN	FIX	S	PFQ	Between subjects
Rottenberg et al. (2007) [21]	AD (860)		19.30	54.18	UC	VA	DIEM	C(1) F(16) NNC(4) NPC(1)	FIL/SHA	GR	RAN	S	PFQ	Within subject
Samson et al. (2015, Experiment 3) [85]	AD (411)		38.51	59.60	Other	VA, AR	DEM	C(1) F(50) NNC(1) NPC(1)	FIL	IN	RAN	S	DIMS DIES	Within subject
Sato et al. (2007) [86]	AD (31)		21.90	51.61	Other	VA, AR	DEM	C(1) F() NNC(4) NPC(2)	SHA	GR	RAN	S	AFFECT GRID	Within subject
Schaefer et al. (2010) [28]	AD (364)		19.60	80.76	UC	VA, AR	DIEM	C(1) F(10) NNC(4) NPC(2)	FIL	GR	RAN	S	PANAS SEA	Within subject
Silvestrini & Gendolla (2007) [87]	AD (43)		24	83.72	UC	VA	DEM	C(3) F(1) NNC(1) NPC(1)	FIL	IN	RAN	S	VAS	Within subject
Stephens et al. (2010) [88]	AD (50)		19.30	54	UC	VA, AR	DEM	C(1) F(13) NNC(3) NPC(2)	SHA	IN	RAN	S	ASR	Within subject
Vianna & Tranel (2006) [89]	AD (16)		26.70	56.25	Other	VA, AR	DEM	C(1) F(10) NNC(3) NPC(1)	DOC	IN	RAN	S	DIMS	Within subject
Vianna et al. (2006) [3]	AD (20)	CD (20)	38.80 38.10	65 55	Other	VA, AR	DEM	C(1) F(10) NNC(3) NPC(1)	DOC	IN	RAN	S	DIMS	Between subjects
Vicente et al. (2009) [90]	AD (16)	PD (26)	56.60 56.65	42.85 34.61	Other	VA	DIEM	C(1) F(6) NNC(4) NPC(1)	Other	IN	FIX	S	DES	Between subjects
Vicente et al. (2011) [91]	AD (15)	PD (33)	57.27 62.33	53.33 66.66	Other	VA	DIEM	C(1) F(6) NNC(4) NPC(1)	Other	IN	FIX	S	DES	Between subjects
Von Leupoldt et al. (2007) [92]	AD (297)		8.7	45.11	Other	VA, AR	DEM	C(1) F(3) NNC(1) NPC(1)	Other	GR	RAN	S	SAM	Within subject
Yuen & Lee (2003) [93]	AD (54)		19	33.33	UC	VA	DEM	C(3) F(1) NNC(1) NPC(1)	FIL	IN	RAN	S	DIMS	Within subject

*Note*. AD = Adults; AF = Affect Grid; AR = Arousal; ASR = Affect Self-Report; BD = Bipolar Disorder; C = Conditions, number of conditions; CONAR = Continuous Affect Ratings; CD = Crohn Disease; DD = Depressive Disorder; DES = Differential Emotions Scale; DEM = Dimensional Emotional Model; DIEM = Discrete Emotional Model; DIES = Discrete Emotions Scale; DIMS = Dimensional Model Scale (measure of valence and/or arousal); DOC = Docummentary; DS = Depressive Symptomatology; DY = Dysphoria; EMOEX = Scale about emotional experience; ERF = Emotion Report Form; ES = Emotions Scale; F = Films, number of films watched by each participant; FIL = Films; FIL/SHA = Films and shapes watched; FIX = Fixed presentation; GR = Group session; IN = Individual session; NNC = Number of Negative Categories; NPC = Number of Positive Categories; OL = Older adults (< 60 years old); PANAS = Positive and Negative Affect Schedule; PA/NA = Positive and Negative Affect Scale; PD = Parkinson Disease; PFQ = Post-Film Questionnaire; PLEIN = measures of Pleasantness and Intensity of emotions; RAN = Random presentation; S = Sound; SAM = Self Assessment Manikins; SEA = Self-reported Emotional Arousal; SHA = Shapes, screen with shapes; SIMC = Six-Item Mood Check; UC = University Community; UNK = Unknown data; VA = Valence; VAS = Visual Analogue Scale; WS = Without Sound.

### Overall Effect Size (Valence and arousal regardless of the type of stimulus)

A total of 178 effect sizes were obtained from 45 publications including 6,675 participants. The effect sizes were independent of each other. The *Q*-test established heterogeneity across the studies (*p* < .001, *I*^*2*^ = 96.61), and the random-effects model was thus used to establish the overall effect size. Hedges’ *g* effect size was found to be -1.49, with a variance of 0.01, 95% *CI* [-1.64, -1.34], *p* < .001.

### Valence ratings with negative and positive stimuli

**Fig 2:**
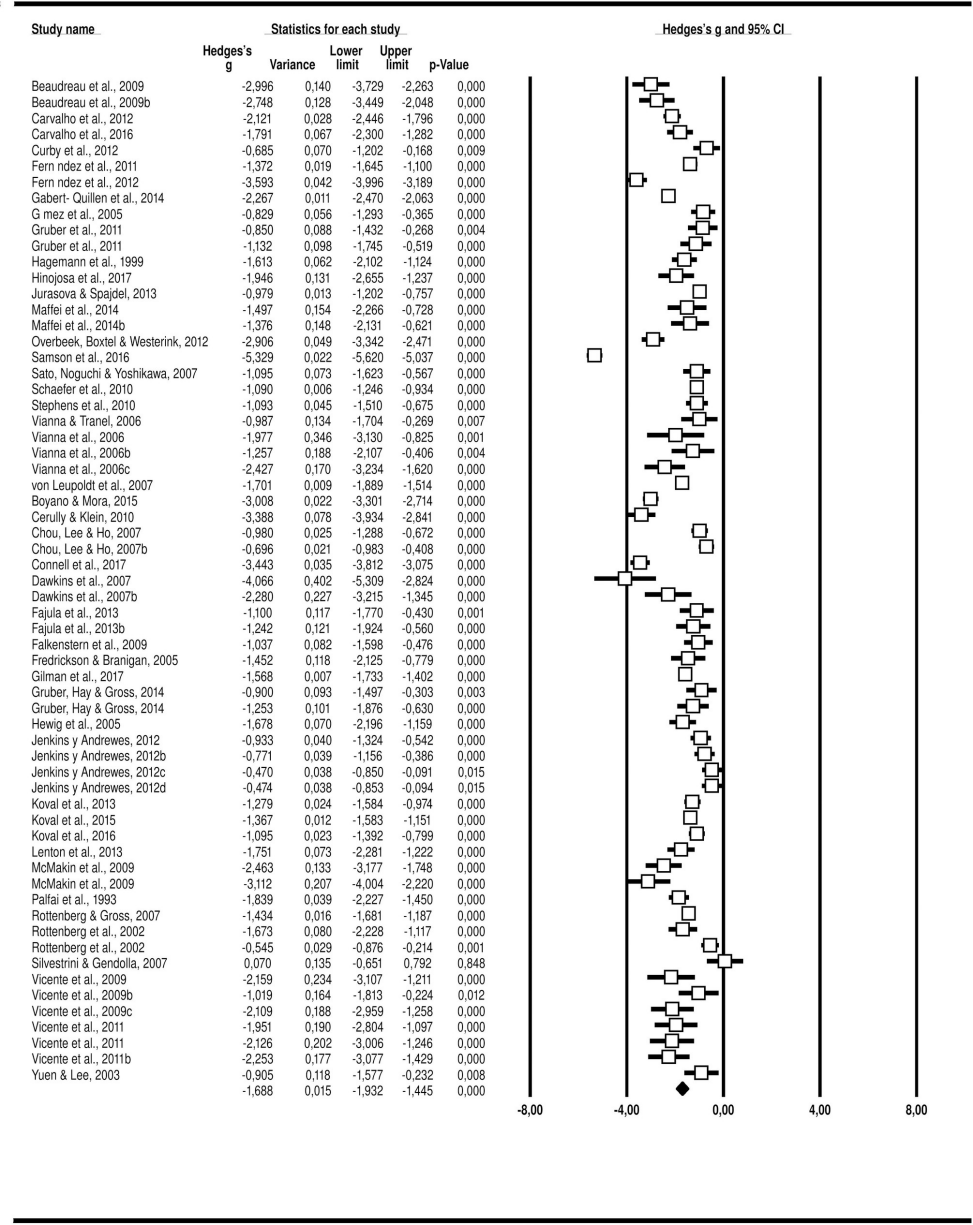
Hedges’ *g* for each study and combined (random-effects model) for valence with negative stimulus.

**Fig 3:**
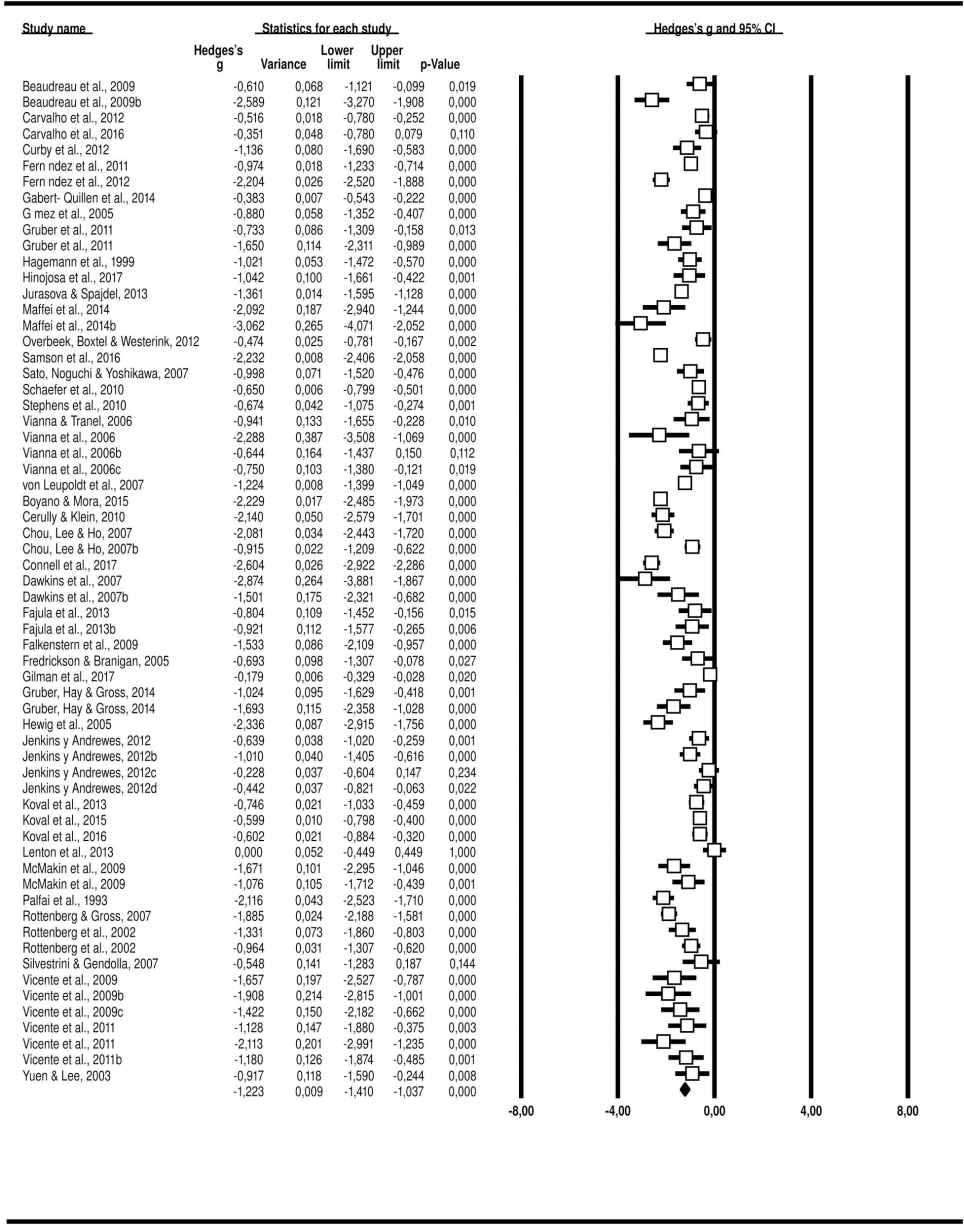
Hedges’ *g* for each study and combined (random-effects model) for valence with positive stimulus.

Based on 45 studies and 6,675 participants, we found 63 effect sizes. For negative stimuli, the *Q*-test established heterogeneity across the studies (*p* < .001, *I*^*2*^ = 95.80), and the random-effects model was thus used to establish the effect size. Hedges’ *g* effect size was found to be -1.69, with a variance of 0.02, 95% *CI* [-1.93, -1.45], *p* < .001 (Fig 2). For positive stimuli, the *Q*-test showed heterogeneity across the studies (*p* < .001, *I*^*2*^ = 93.74), and the random-effects model was used to establish the effect size. Hedges’ *g* effect size was found to be -1.22, with a variance of 0.01, 95% *CI* [-1.41, -1.04], *p* < .001 (Fig 3).

### Arousal ratings with negative and positive stimuli

**Fig 4:**
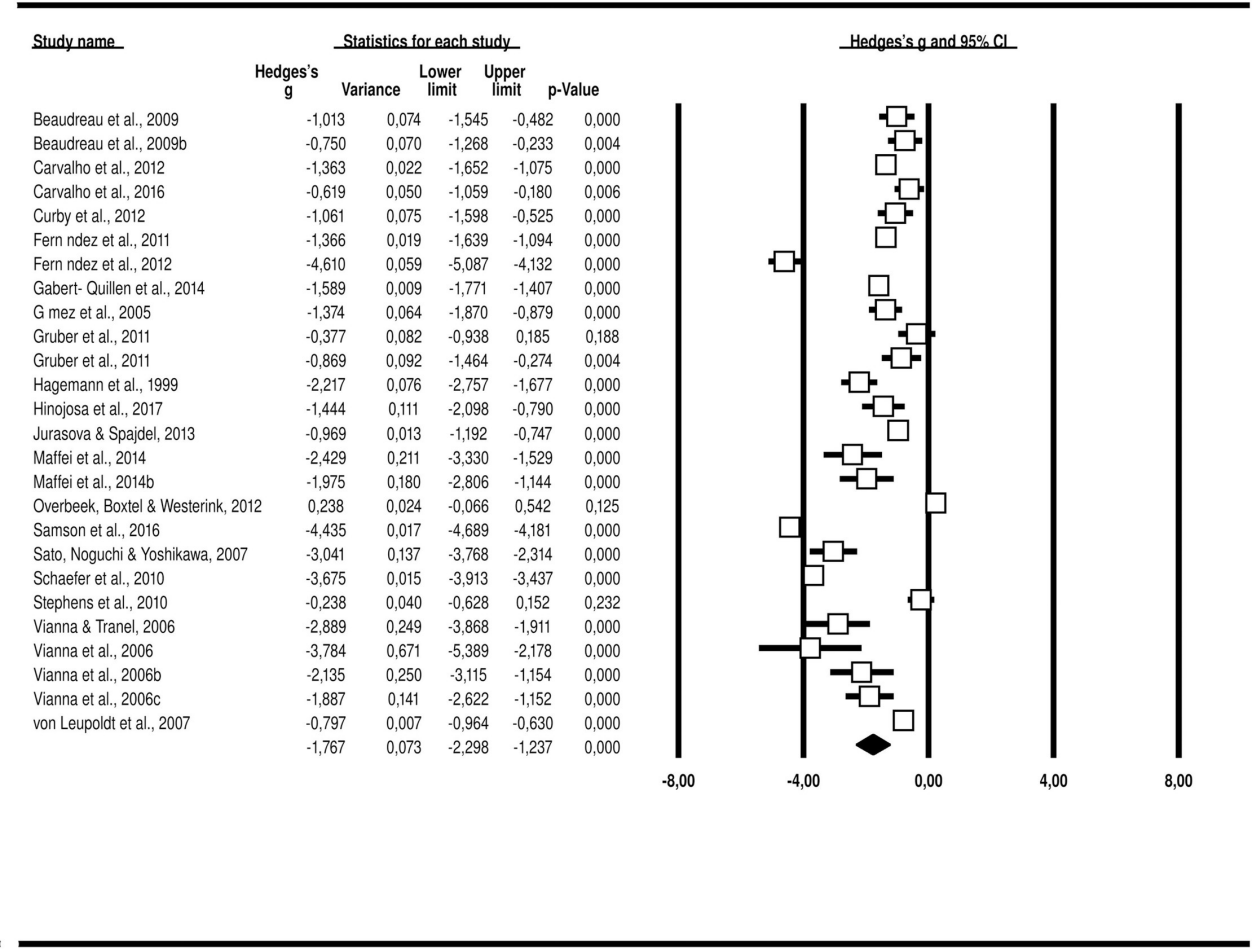
Hedges’ *g* for each study and combined (random-effects model) for arousal with negative stimulus.

**Fig 5:**
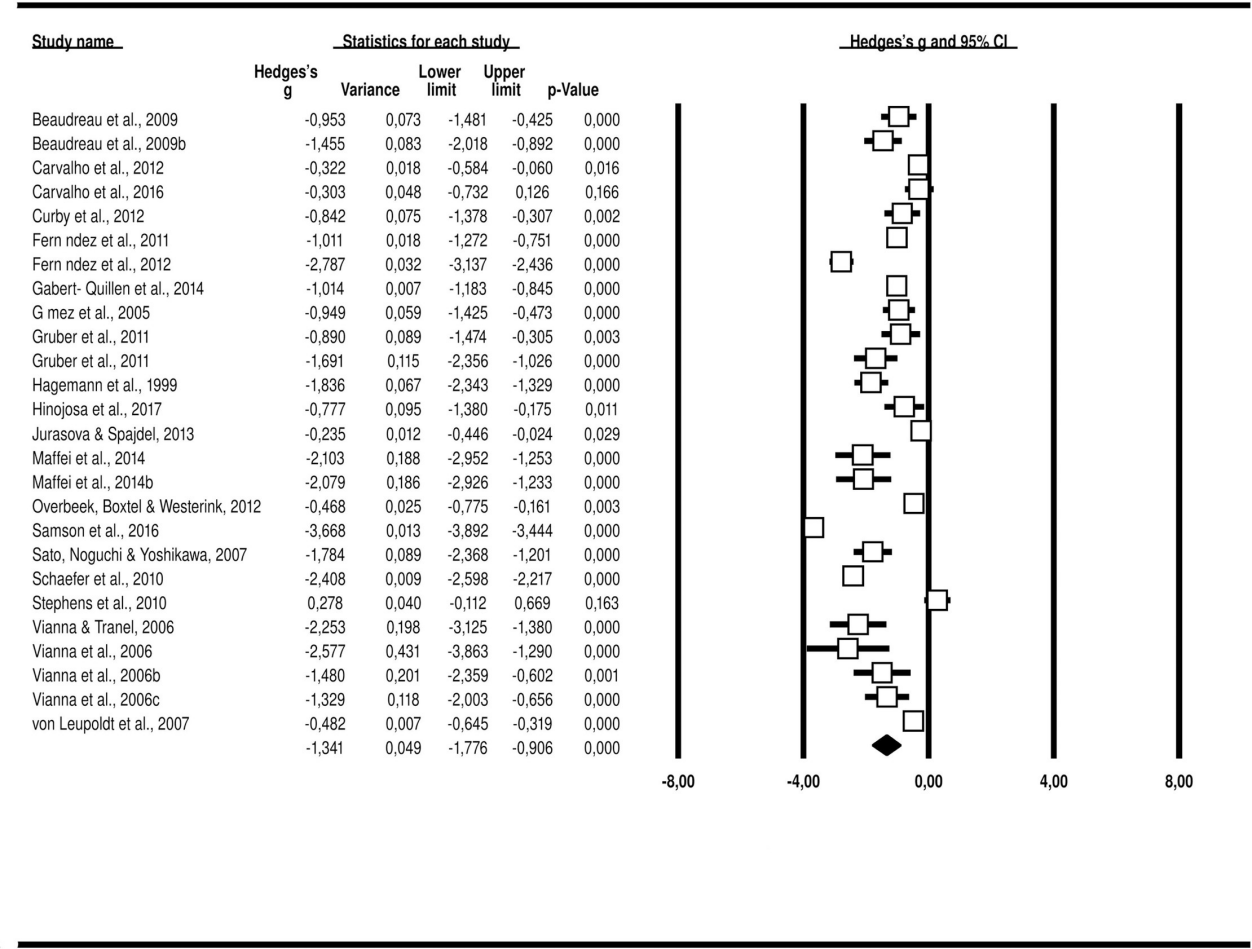
Hedges’ g for each study and combined (random-effects model) for arousal with positive stimulus.

Based on 21 studies and 2,625 participants, we found 26 effect sizes. For negative stimuli, the *Q*-test showed heterogeneity across the studies (*p* < .001, *I*^*2*^ = 98.13), and the random-effects model was used to establish the effect size. Hedges’ *g* effect size was found to be -1.77, with a variance of 0.07, 95% *CI* [-2.30, -1.24], *p* < .001 (Fig 4). For positive stimuli, the *Q*-test showed heterogeneity across the studies (*p* < .001, *I*^*2*^ = 97.59), and the random-effects model was thus used to establish the effect size. Hedges’ *g* effect size was found to be -1.34, with a variance of 0.05, 95% *CI* [-1.78, -0.91], *p* < .001 (Fig 5).

### Evaluation of moderators

To determine the variables that might have an effect on heterogeneity, we conducted a meta-regression with all the possible influencing variables. We found that the number of positive and negative categories in the films was statistically significant (*Q*_*R*_ = 13.80, *p* = .541, *I*^*2*^ *=* 95.14, *R*^*2*^ = 0.00, number of studies = 62) for negative valence. For positive valence, we found that using the combined option of shapes screensaver and film clips as a neutral stimulus was statistically significant (*Q*_*R*_ = 12.64, p = .630, *I*^*2*^ = 90.54, *R*^*2*^ = 0.10, number of studies = 62). We found that the number of films was statistically significant (QR = 14.87, p = .387, I^*2*^ = 96.29, R^*2*^ = 0.24, number of studies = 26) for negative arousal. Finally, we found no statistically significant variables (*Q*_*R*_ = 8.09, p = .885, *I*^*2*^ = 96.20, *R*^*2*^ = 0.00, number of studies = 26) for positive arousal. Tables 2–5 show the results for these combined models.

**Table 2:** Linear regression model for the response on valence with negative stimuli.

Covariate	Coefficient	Standard	95%	95%	2-sided
		Error	Lower	Upper	*p*-value
**Intercept**	-0.94	1.34	-3.56	1.68	.482
**Popular films NS**	0.54	0.38	-0.21	1.28	.157
**Other NS**	-0.09	0.50	-1.07	0.89	.858
**Shapes NS**	-0.31	0.49	-1.26	0.65	.527
**Shapes and popular films NS**	-0.11	0.67	-1.43	1.20	.866
**Number of films**	-0.01	0.01	-0.04	0.01	.364
**% women**	-0.01	0.02	-0.04	0.02	.512
**Individual session**	0.27	0.39	-0.49	1.03	.490
**Sound**	-0.32	0.61	-1.53	0.88	.601
**PosNegFilms**	0.33	0.16	0.01	0.65	.043
**Random**	-0.19	0.47	-1.11	0.72	.681
**University students**	0.02	0.38	-0.71	0.76	.955
**More than 1 condition**	-0.91	0.49	-1.86	0.04	.061
**Older adults**	-0.33	0.59	-1.48	0.82	.579
**Emotional disorders**	0.80	0.65	-0.48	2.08	.222
**Dimensional model**	-0.10	0.34	-0.77	0.57	.767

*Note*. (NS) = Neutral stimulus; PosNegFilms = Number of positive and negative categories in films.

**Table 3:** Linear regression model for response on valence with positive stimuli.

Covariate	Coefficient	Standard	95%	95%	2-sided
		Error	Lower	Upper	*p*-value
**Intercept**	-2.21	0.85	-3.87	-0.56	.009
**Popular films NS**	-0.38	0.25	-0.87	0.10	.124
**Other NS**	-0.44	0.34	-1.11	0.22	.194
**Shapes NS**	-0.59	0.33	-1.23	0.05	.072
**Shapes and popular films NS**	-1.14	0.44	-2.01	-0.28	.009
**Number of films**	0.01	0.01	-0.01	0.03	.388
**% women**	0.00	0.01	-0.02	0.02	.855
**Individual session**	0.23	0.26	-0.29	0.74	.387
**Sound**	0.70	0.41	-0.11	1.51	.089
**PosNegFilms**	0.25	0.17	-0.08	0.58	.134
**Random**	0.11	0.32	-0.50	0.73	.717
**University students**	0.17	0.26	-0.35	0.69	.517
**More than 1 condition**	0.04	0.29	-0.53	0.62	.882
**Older adults**	-0.01	0.38	-0.77	0.74	.974
**Emotional disorders**	-0.04	0.43	-0.89	0.80	.924
**Dimensional model**	0.03	0.23	-0.42	0.48	.898

*Note*. (NS) = Neutral stimulus; PosNegFilms = Number of positive and negative categories in films.

**Table 4:** Linear regression model for response on arousal with negative stimuli.

Covariate	Coefficient	Standard	95%	95%	2-sided
		Error	Lower	Upper	*p*-value
**Intercept**	0.98	2.77	-4.45	6.41	.724
**Popular films NS**	0.44	0.91	-1.35	2.22	.632
**Other NS**	-0.37	1.39	-3.10	2.36	.793
**Shapes NS**	0.55	0.91	-1.23	2.34	.542
**Shapes and popular films NS**	0.10	1.22	-2.29	2.49	.933
**Number of films**	-0.09	0.04	-0.16	-0.02	.013
**% women**	-0.05	0.03	-0.12	0.02	.135
**Individual session**	0.57	0.84	-1.08	2.22	.498
**Sound**	0.37	1.13	-1.85	2.59	.741
**PosNegFilms**	-0.43	0.31	-1.03	0.18	.166
**University students**	0.98	0.79	-0.57	2.53	.214
**More than 1 condition**	0.75	1.60	-2.39	3.89	.640
**Older adults**	1.10	1.56	-1.96	4.16	.481
**Emotional disorders**	1.04	1.66	-2.21	4.29	.529
**Dimensional model**	0.30	1.20	-2.04	2.65	.799

*Note*. (NS) = Neutral stimulus; PosNegFilms = Number of positive and negative categories in films.

**Table 5:** Linear regression model for response on arousal with positive stimuli.

Covariate	Coefficient	Standard	95%	95%	2-sided
		Error	Lower	Upper	*p-*value
**Intercept**	-1.07	2.61	-6.19	4.06	.683
**Popular films NS**	-0.17	0.85	-1.84	1.51	.847
**Other NS**	0.08	1.19	-2.26	2.41	.948
**Shapes NS**	0.04	0.86	-1.65	1.73	.963
**Shapes and popular films NS**	-0.36	1.21	-2.73	2.01	.765
**Number of films**	-0.05	0.03	-0.12	0.01	.090
**% women**	-0.03	0.03	-0.09	0.04	.427
**Individual session**	0.22	0.78	-1.31	1.75	.778
**Sound**	0.97	1.10	-1.19	3.13	.377
**PosNegFilms**	0.10	0.35	-0.58	0.78	.770
**University students**	0.74	0.77	-0.77	2.25	.340
**More than 1 condition**	0.40	1.53	-2.59	3.39	.792
**Older adults**	0.75	1.49	-2.17	3.67	.617
**Emotional disorders**	0.25	1.59	-2.87	3.37	.874
**Dimensional model**	-0.04	1.07	-2.14	2.05	.968

*Note*. NS = Neutral stimulus; PosNegFilms = Number of positive and negative categories in films.

### Evaluation of publication bias

We examined the publication bias for negative valence, finding none, with Egger’s test (*p* = -.281) yielding a statistically non-significant result. However, Egger’s test (*p* = .026) was statistically significant, suggesting the presence of a publication bias for positive valence.

In the evaluation of negative arousal, no publication bias was found, with Egger’s test (*p* = .376) for negative arousal and with Egger’s test (*p* = .385) for positive arousal yielding statistically non-significant results. S1–S4 Figs shows the funnel plots of the effect size for the assessment of valence and arousal with negative and positive stimuli.

## Discussion

The overall aim of this meta-analysis was to provide theoretical and practical information for researchers who decide to use this method of emotional induction in their research. We examined the mean effects of film mood induction for positive and negative mood states. The results of our meta-analytical integrations revealed large effect sizes for both negative and positive induction using film clips MIP. These results are described in the following sections.

### Differences in induction using negative and positive stimuli

Although scientific research suggests a variety of MIPs are useful for inducing positive and negative emotions, previous literature reviews tend to highlight the effectiveness of film clips [4, 5, 6, 22]. Hence, we wished to examine the mean effects of positive and negative emotional targets using film clips. Based on the results of 63 effects sizes for valence and 21 for arousal, our results show that the effectiveness of both positive and negative induction is significantly high. Although a direct comparison between negative and positive emotional induction cannot be computed, it can be observed that negative induction presents a larger effect size for both affective valence and level of arousal. Previous reviews have shown that negative mood induction is more powerful than positive emotional induction [5, 6]. These results might be explained by the level of motivation in participating in the studies. Thus, if the general mood state during the experiment is positive, the difference between this state and the state obtained by the positive emotional target will be low [5]. The difference in effectiveness between positive and negative induction might also be explained by the neutral stimuli, which are used to determine the baseline state prior to the induction process. The neutral stimuli used tend to encourage relaxation because they involve calming actions such as listening to peaceful music, breathing exercises or viewing a nature documentary (e.g., [80, 91]). Sweeney [94] defines relaxation as “a positively perceived state or response in which an individual feels relief of tension or strain”. Therefore, the differences between a positive emotional state and a neutral state could be less significant than those between the same neutral state and the affective state after the negative induction.

Last, it should be considered that these findings may be the result of the affect measurement. The present meta-analysis reviewed studies that assess positive and negative affect via self-report. A basic consideration in self-reports is the subjective interpretation of cues from the context, their physiological sensations and the cognitive information about their current mood [95]. The literature suggests that the processing of affective stimuli is faster when participants respond to a negative high-arousal stimulus or to a positive low-arousal stimulus [96, 97]. Thus, our results may be explained by a question of developmental survival. Emotion research argues that humans process positive information and negative information differently. Specifically, negative information has a stronger psychological impact than positive information [98], requiring greater attention and being recognized more accurately [99]. From a developmental approach, it may be considered that processing of negative information is more potent than positive information because it is directly linked to survival. Negative emotions are associated with the activation of the defense system [100]. Our results indicate a larger effect size for negative valence and arousal than for positive valence and arousal. Self-report forms were completed immediately after each induction. In order to give meaning, attend and respond to the stimuli around us, our energy levels must be high, and we need to be active during and immediately after exposure to the inductive stimuli. This might explain the large effect sizes of the negative dimensions.

### Determinants of the strength of affective reaction

Film clips are the most commonly used stimuli in mood induction [22], and are currently one of the most widely recognized and accepted MIPs. However, researchers might have doubts when selecting the type of sample or the most adequate methodological procedure, given that the previous literature presents diverse findings on both aspects. The strength of the MIP is arguably directly related to the population selected for research, the experimental procedure used or the characteristics of the audiovisual stimuli. Accordingly, the present meta-analysis was also designed with the aim of answering such practical questions and thus provide guidelines on emotion induction research.

#### Affective reactions by sample

Regarding the characteristics of the sample, our results show no variables are related to the strength of emotional induction. With respect to gender, previous literature reviews have reported that participants” gender is unrelated to the effect size obtained [5]. In this sense, the present meta-analysis also finds no evidence that gender influences the strength of induction of positive and negative emotions using film clips as the MIP. As for age, the literature provides evidence on changes in emotional response over the adult lifespan (young adults vs. older adults). Moreover, older adults have been found to exhibit reduced reactivity to negative stimuli [56]. In addition, previous studies have used film clips as a MIP with this population, with adequate rates of success in inducing positive and negative moods (e.g., [61, 64]). In the same line, this meta-analysis finds no evidence that age affects the strength of mood induction. Film clips appear to be an effective method for mood induction in both young and older adults. Nonetheless, most of the participants in the studies included in this review are young adults. The same is true of the comparison between clinical and non-clinical population. Despite the low level of non-clinical populations in this work, the results suggest there exist no differences between clinical and non-clinical populations that might affect the strength of mood induction. Hence, film clips are apt for mood induction procedures in clinical population. Finally, we considered possible differences between college students and community members, supposing that the former might exhibit a stronger response to mood induction. This hypothesis emerges given that the familiarity of students with experimental tasks in university settings might lead them to experience the demand effect more than other participants. However, the present review has found no evidence of such effects, as is the case in previous reviews [22].

In summary, sample-related variables, such as age, gender composition, sample community and clinical disorders seem to have no impact on the effectiveness and strength of emotion induction using film clips.

#### Affective reactions according to the research procedure

Regarding the influence of the different variables considered in the experimental procedures, the present meta-analysis shows the need to consider the characteristics of the stimuli, film clips in this case, since the way they are used may affect the strength of the mood induction generated in the procedure.

With regard to the induction of negative emotions, it was observed that the number of film clips and emotional categories had an impact on the strength of the affective reaction. Specifically, it was found that the larger the number of emotional categories elicited and the greater the number of film clips used, the greater is the impact on the strength of the mood induction. These findings might be explained by the accumulative effect of mood states, or, in other words, excitation transfer. Take the emotion of anger, for example. One way to evoke anger is to use only one film clip. However, if the goal is to induce different negative states, we will choose several film clips, one for anger and other clips for the remaining negative emotions. In a short period of time, watching only one film clip is a different experience compared to watching several film clips. The strength of mood induction is likely to be greater in the second case. The larger the number of films or emotional categories, the longer is the time of exposure to emotional stimuli. When one emotional state after another is induced in a person, the baseline state will not normally be recovered in the period between one stimulus and another. Considering the dimensions of valence and arousal, this phenomenon is dependent on the latter dimension, and thus the effect of residual arousal is posited. The excitation transfer theory [101, 102] is based on the fact that when exposure to a first stimulus finishes, the physiological arousal does not suddenly stop. Sympathetic activation persists for a certain time and declines slowly, potentially impacting the effect of the subsequent induction stimuli.

The fact that both the number of emotional categories and the number of film clips used affects the induction of negative emotions but not that of positive ones might be a consequence of negative information being processed differently from positive information. Indeed, negative information is thought to be more informative and its recognition is more robust and intense than that of positive information as it is considered a developmentally more adaptive process. Ignoring negative information (e.g. a danger stimulus) may put one’s survival at risk [98, 99]. Hence, it is unsurprising that the results in this review show that continued exposure to negative stimuli affects the strength of mood induction. This finding is interesting as it supports the need to establish a rest period between stimuli (e.g., the use of distraction tasks) that facilitates emotional recovery when an experiment includes more than one mood induction. In addition, it highlights the need to control for the order of the stimuli based on their affective valence. In an experimental procedure, inducing negative emotions before positive ones could have an impact on the emotion induction obtained in the latter.

Finally, with regard to the induction of positive emotions, it was observed that the characteristics of neutral stimuli influence the strength of the mood induction. For this reason, it is worth noting the importance of selecting an appropriate neutral stimulus to establish the baseline and of using the emotional responses to this stimulus as a control variable. The present review shows the existence of a diversity of stimuli or techniques used for neutral induction. Moreover, the importance of the choice of the neutral stimulus in mood induction procedures is often neglected. An inappropriate selection of the neutral stimulus may have an impact on the effect of the emotional stimuli on participants and even on the capacity for recovery following the induction. In this sense, our results suggest that the combined use of neutral clips from popular films and a shapes screensaver is the most effective stimulus. The findings also seem to show that the fact the neutral stimuli share the same idiosyncratic characteristics as the other emotional clips facilitates the experimental procedure in mood induction [59]. In this case, the combination of both stimuli is presented audiovisually, encouraging coherence across the experimental session.

## Limitations and future research

This meta-analysis suffers from a number of limitations. First, to assess the emotional response evoked by the induction method, some studies have examined valence (positive, negative and neutral) and arousal (high, medium and low), while others have considered the type of emotion (e.g., disgust, anger, fear and sadness as negative emotions). This complicates a direct comparison of these studies. Second, it was not possible to assess the differences within each emotional category (e.g., gender differences, taking into account the approach and avoidance models) due to the conflictive classification of these emotions. Third, the fact that no association was found between the emotional response and type of sample (for example, age and clinical population) may have been due to the lack of statistical power of the studies selected that included these moderator variables. Clear evidence of the effect of these variables would require further studies with older adults and clinical population with a variety of disorders.

For future experimental research, we recommend investigating whether film clips are useful for the study of other emotional areas. In this study, we examined subjective experience to understand the emotional process. Future studies could review the implication of the use of films for other dimensions, for example, from a neuroscientific perspective. In addition, it could be interesting for future reviews to study film clip MIPs to induce discrete mood states at both physiological and subjective levels. In relation to the aforementioned discrete emotion model, it would also be necessary to improve the definition of positive emotions and the number of positive emotions used in the MIPs, because there is a lack of consensus, as the present review reflects. It would also be of interest to determine whether results vary according to whether the measurement is conducted after or during the viewing of the clips. It would also be interesting to increase the study of MIPs, especially in older adults, to determine whether they respond differently to young adults both with regard to the emotional reaction generated by the audiovisual stimuli and their subsequent recovery. In this sense, it might also be useful to study how the changes in the aesthetics and contemporaneity of films affect the emotional reaction of individuals of different generations.

## Conclusions

Although work remains to be done on classifying and enhancing our understanding in the field of emotion psychology, and more specifically on the use of audiovisual techniques in the laboratory, our meta-analysis suggests that mood induction by film clips is a highly effective method to generate negative and positive affective reactions. All the effect sizes on mood induction using film clips, both in terms of valence and arousal, were large, ranging between -1.22 and -1.77. Moreover, this quantitative review highlights the need to take into account the variables related to the experimental procedure since these may directly affect the strength of the mood induction obtained. Findings suggest that this effect may vary according to whether negative or positive emotions are being elicited. In the case of negative emotions, the number of emotional categories evaluated and the number of film clips used in the procedure may contribute to the strength of the mood induction. Furthermore, the type of stimulus used to measure the baseline state appears to influence the strength of the induction of positive emotions.

Despite some inconsistencies among individual studies examined, this work provides information on the advantages and disadvantages of using this engaging and increasingly popular methodology, encouraging further research to enhance the understanding of the complex emotional system and its functioning.

## Supporting information

S1 Fig Funnel plot of standard error by hedges’ *g* for valence with negative stimulus. (TIF)

S2 Fig Funnel plot of standard error by hedges’ *g* for valence with positive stimulus. (TIF)

S3 Fig Funnel plot of standard error by hedges’ *g* for arousal with negative stimulus. (TIF)

S4 Fig Funnel plot of standard error by hedges’ *g* for arousal with positive stimulus. (TIF)

S1 Dataset Dataset of the study. (CMA)
