# Supplementary figures and images for: How effective are films in inducing positive and negative emotional states? A meta-analysis
Source: PLoS One. 2019 Nov 21;14(11):e0225040. doi: 10.1371/journal.pone.0225040 (PMC6872151; doi:10.1371/journal.pone.0225040)

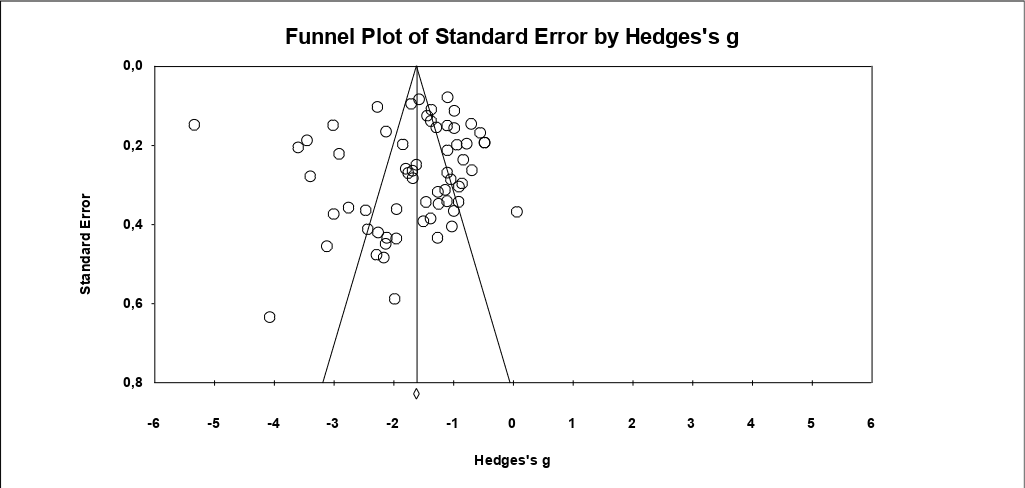

Supplement: S1 Fig — (TIF) [file pone.0225040.s001.tif]

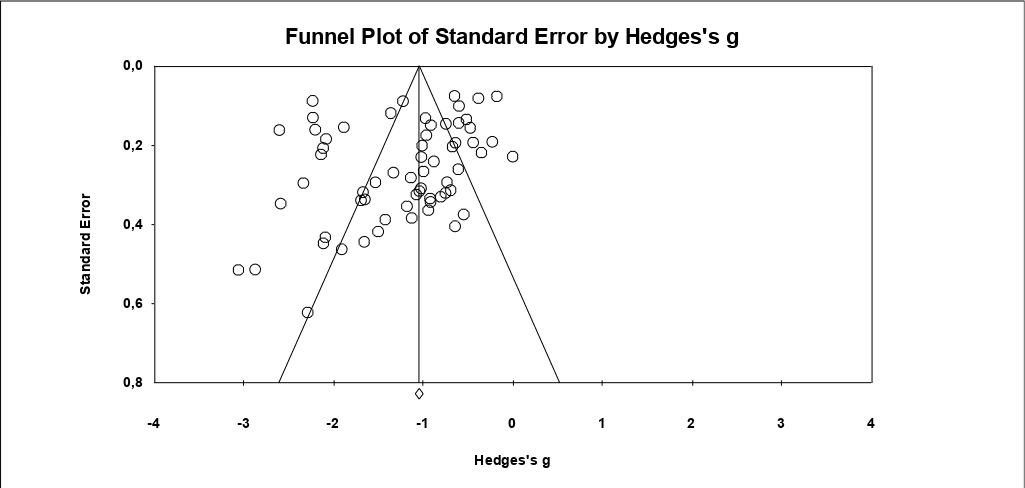

Supplement: S2 Fig — (TIF) [file pone.0225040.s002.tif]

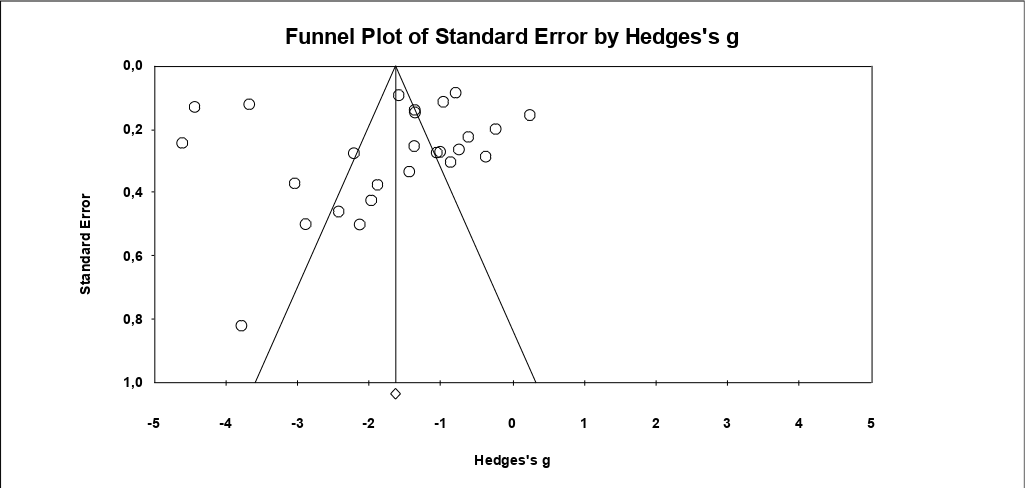

Supplement: S3 Fig — (TIF) [file pone.0225040.s003.tif]

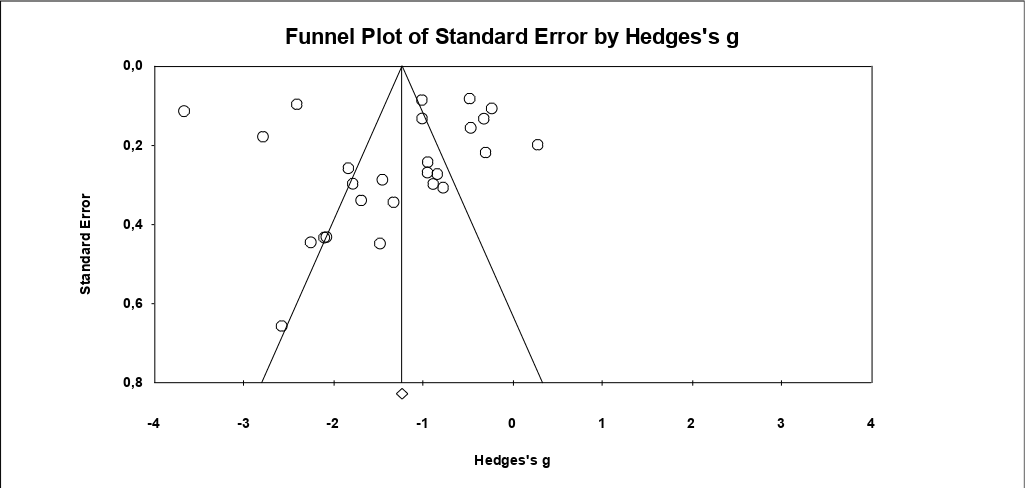

Supplement: S4 Fig — (TIF) [file pone.0225040.s004.tif]
